# Regional contribution of vascular dysfunction in white matter dementia: clinical and neuropathological insights

**DOI:** 10.3389/fneur.2023.1199491

**Published:** 2023-06-16

**Authors:** Jonathan Pansieri, Gina Hadley, Andrew Lockhart, Marco Pisa, Gabriele C. DeLuca

**Affiliations:** Nuffield Department of Clinical Neurosciences, University of Oxford, Oxford, United Kingdom

**Keywords:** white matter dementia, vascular dementia, multiple sclerosis, Alzheimer’s disease—AD, vascular dysfunction

## Abstract

The maintenance of adequate blood supply and vascular integrity is fundamental to ensure cerebral function. A wide range of studies report vascular dysfunction in white matter dementias, a group of cerebral disorders characterized by substantial white matter damage in the brain leading to cognitive impairment. Despite recent advances in imaging, the contribution of vascular-specific regional alterations in white matter dementia has been not extensively reviewed. First, we present an overview of the main components of the vascular system involved in the maintenance of brain function, modulation of cerebral blood flow and integrity of the blood–brain barrier in the healthy brain and during aging. Second, we review the regional contribution of cerebral blood flow and blood–brain barrier disturbances in the pathogenesis of three distinct conditions: the archetypal white matter predominant neurocognitive dementia that is vascular dementia, a neuroinflammatory predominant disease (multiple sclerosis) and a neurodegenerative predominant disease (Alzheimer’s). Finally, we then examine the shared landscape of vascular dysfunction in white matter dementia. By emphasizing the involvement of vascular dysfunction in the white matter, we put forward a hypothetical map of vascular dysfunction during disease-specific progression to guide future research aimed to improve diagnostics and facilitate the development of tailored therapies.

## 1. Introduction

The term white matter (WM) dementia refers to a clinical syndrome characterized by dysexecutive symptoms, such as slowed processing speed and impaired sustained attention, due to multifocal or diffuse WM damage (1). Despite their high prevalence across several conditions, WM involvement in dementia has been historically overshadowed by a focus on gray matter (GM) pathology that typically predominates in cortical dementia syndromes, such as Alzheimer’s disease (AD). However, recent advances in neuroimaging have shed light on the intimate relationship between WM and cortical gray matter (CGM) and deep gray matter (DGM) regions, and how they govern vital functions, such as emotion and memory. The causes of WM dementia remain elusive, although vascular dysfunction has emerged as a key early pathophysiological contributor (2–14). Indeed, the maintenance of adequate blood supply and vascular integrity is fundamental to ensure brain function in health. In WM dementia, growing evidence implicates vascular alterations, such as aberrant angiogenesis (7) and blood–brain barrier (BBB) impairment, and their impact on cerebral blood flow (CBF), as important culprits. Altogether, these abnormalities impact homeostasis (8, 9), brain metabolism (10, 11), and glial activation (12, 13), which contribute to neurodegeneration and the dementia syndromes that ensue. Although it is known that various neurological diseases are influenced by specific regional vascular deficits within the brain (15, 16), this angle has been poorly explored in the spectrum of WM dementia. Thus, a better understanding of the relationships, connections and differences between WM and GM vasculature may help to decipher the causes and consequences of WM dementia and are the focus of the current review.

After presenting the main components of the vascular system involved in brain maintenance in health and normal aging, we review the regional contribution of vascular changes to WM pathology (see anatomical considerations in Table 1) in the pathogenesis of three distinct conditions: the archetypal white matter predominant neurocognitive dementia that is vascular dementia, a neuroinflammatory predominant disease (multiple sclerosis, MS) and a neurodegenerative predominant disease (AD). Finally, we consider factors that contribute to vascular changes in vascular dementia, MS and AD. In so doing, we hope to highlight the importance of WM pathology in the pathogenesis of the spectrum of dementia types to guide future research.

**Table 1:** Neuroanatomical considerations.

White matter (WM)	**WM** consists of subcortical tissues, containing myelinated axons which connect neurons and glial cells. It represents almost half of the brain’s volume. The **corpus callosum** is the largest WM structure in the brain that connects the left and right hemisphere. WM is an essential component connecting areas of gray matter (GM) throughout the CNS and coordinates their communications.
Cortical gray matter (CGM)	The CGM (or **cerebral cortex**) is the outermost tissue of the brain lying on the top of the cerebrum. It is made up of folded GM defined as gyri (top areas) and sulci (deep areas). It covers subcortical WM and is divided into four lobes: **frontal, parietal** (where **precuneus** is the portion of the superior parietal lobe), **temporal and occipital**. The **cingulate cortex** located in the medial aspect of the cerebral cortex wraps the corpus callosum. The cerebral cortex contains a highly complex network of glial cells, neurons, axons and extracellular matrix proteins, and is involved in numerous functions such as emotion, memory, learning and language.
Deep gray matter (DGM)	The DGM consists of the thalamus, hippocampus, nucleus accumbens and basal ganglia. It is made up of neurons from which originate deep nerve fibers. **The hippocampus** is a simplified cortical structure located in the medial region of the temporal lobe, and consists of the dentate gyrus surrounded by corpus-ammonis and the subiculum. It has direct connections with the **entorhinal cortex**, and the amygdala. The hippocampus plays a major role in learning and memory. **The thalamus** is located near the center of the brain, including the **hypothalamus**, the subthalamus and the epithalamus. It has connections with structures of the limbic system (hippocampus, amygdala, cingulate cortex) and the cerebral cortex. The thalamus is a crucial gateway which relay the information in the CNS, playing a role in sense processing, spatial learning and memory. **The basal ganglia** is located deep within the cerebral hemispheres. It consists of a group of subcortical nuclei composed of the corpus striatum, the globus pallidus and the substantia nigra. These structures are connected to various brain areas, receiving many inputs from the cerebral cortex and the thalamus. Due to its large connectivity, the basal ganglia is involved in motor control, as well as rule-based learning, working memory and cognitive functions. **The amygdala** is located in the medial temporal lobe in front of the hippocampus. Its main connections include the hippocampus, the basal ganglia through the striatum and the cerebral cortex. The amygdala is primarily responsible of the processing and memorizing of emotional reactions.

## 2. The vascular system in normal brain

**Figure 1:**
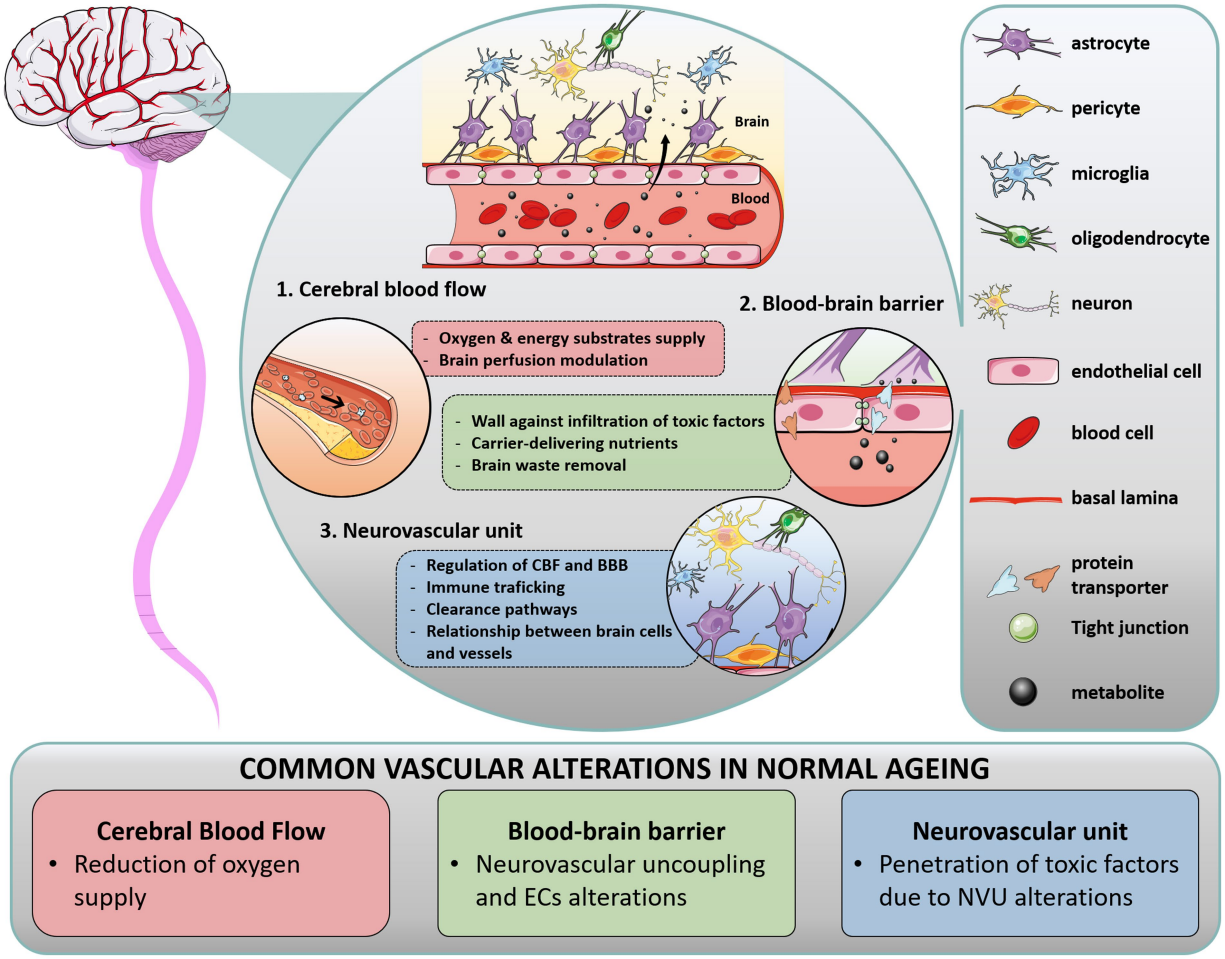
Overview of cerebral vasculature in health and normal aging. In the brain, multiple interactions between vascular structures in the blood–brain barrier (BBB) and neurovascular unit (NVU) regulate cerebral blood flow (CBF). The BBB is a range of unique properties of endothelial cells and extracellular structures apposed to the membrane of these endothelial cells, forming the basal lamina. At the interface of the peripheral circulation and brain parenchyma, the NVU is composed of various cells, such as glial cells (astrocytes end-feet in close association with vessel wall, microglia, oligodendrocytes), neurons, and perivascular pericytes, which act together to regulate CBF and clearance pathways. Importantly, blood supply matches local neuronal demand by a mechanism called neurovascular coupling, wherein activated neurons release specific effectors which activate astrocytes and pericytes, inducing the release of vasoactive mediators and subsequently regulating local CBF. In addition to their critical role in brain homeostasis, these structures form a physical barrier protecting the brain against immune infiltrates (macrophages, lymphocytes) and infiltrating pathogens, restraining the entry of harmful molecules (toxins), organisms (bacteria), and infectious agents (viruses). During aging, each component of the cerebral vasculature is impacted.

Blood vessels form a vital infrastructure for the supply of metabolites, oxygen and nutrients throughout the brain. The tubular structure of vessels is formed by vascular endothelial cells (ECs), surrounded by pericytes, astrocytes end-feet and extracellular matrix (ECM, forming the basal lamina), supplemented by direct interaction with neuron processes and oligodendrocytes. In that context, the term “blood–brain barrier” (BBB) encompasses a range of unique properties of brain blood vessels. The BBB tightly regulates cells, molecules and ions trafficking in and out of the parenchyma, maintaining adequate CBF and vessel permeability to ensure neuronal activity and protection against pathogens. The BBB is not a single entity: it is composed of ECs interacting with a network of brain cells (as mentioned above), that orchestrate vascular function with the activity and metabolic needs of the surrounding brain areas, termed the neurovascular unit (NVU). In this section, we briefly describe the function of these key anatomic structures in healthy brain (Figure 1) and their alteration in normal aging, commenting on the differences observed in WM and GM areas.

### 2.1. Vascular function in healthy brain and normal aging

#### 2.1.1. Cerebral blood flow

By convention, CBF is defined as the volume of blood which flows at a rate of delivery through a defined quantity of brain tissue during a specific period of time (17). The brain needs an anatomically disproportionate oxygen supply, representing more than 20% of the total oxygen in the body while accounting for only 2% of the total body weight (18). Thus, CBF must modulate brain perfusion in order to supply oxygen and energy substrates essential for baseline neurological function. When neuronal activation occurs, an increase in oxygen and energy demand is followed by an increase in CBF via arterial vasodilation to supply essential metabolites, wherein glucose and its surrogates (the primary fuel source for the brain) enter the brain to complete the aerobic glycolysis (19).

With aging, cerebral oxygenation is commonly reduced. However, it is thought that global CBF remains relatively preserved (20). This stability is ensured by homeostatic processes which modulate CBF in response to cerebral perfusion pressure changes and various external and internal physiological factors, such as vasoactive stimuli and neuronal activity changes (21–27).

#### 2.1.2. The blood–brain barrier

##### 2.1.2.1. Endothelial cell as the wall of the vessels

ECs form an efficient physiological wall ensuring vessel integrity. ECs are adjoined to each other by tights junctions (TJs), which modulate the paracellular transport of molecules through the BBB, and are composed of a large panel of transmembrane proteins involved in permeability regulation (claudin family) and its maintenance (occludin and Zo-1) (28). In addition, ECs modulate transcellular transport thanks to a wide range of receptor and carrier-mediated transporters, regulating the passage of ions, nutrients, energy substrates and cells (29–33).

With aging, senescence of EC combined with TJ alterations may occur, increasing BBB permeability and neurovascular uncoupling, subsequently impacting the inflammatory milieu and neuronal dysfunction (34).

##### 2.1.2.2. The neurovascular unit

The NVU is an anatomical and functional unit encompassing glial cells (astrocytes end-feet, microglia and oligodendrocytes), mural cells (smooth muscle cells and pericytes) and neuronal processes. It plays a critical role in modulating transport of vasoactive agents and neuromodulators, helping to maintain the cerebral micro-environment and BBB integrity (35). Importantly, the NVU triggers vasodilation/vasoconstriction to match the local energy demand given tight interactions between neuronal processes and vessel-related cells (36). To this end, each type of cell forming the NVU modulates a wide range of dynamic processes. Astrocytes and pericytes structurally support the BBB via their end feet and processes, respectively. Astrocytes cover 90% of the micro-vessel surface (5) and regulate homeostasis processes and neuronal activity, while pericytes have functions in CBF regulation, angiogenesis, protein clearance, and neuroinflammatory mechanisms. Microglia, which also act in concert with astrocytes, are the macrophages of the brain involved in the inflammatory response. Microglial cells interact with neurons to maintain their activity and play an important role in synaptic plasticity. Oligodendrocytes contribute to brain homeostasis by maintaining and producing myelin in both GM and WM.

The structures that form the NVU are intricately linked. With aging, each component of the NVU undergoes substantial changes, resulting in increased BBB permeability and penetration of toxic factors into the brain (37, 38). Dysregulated mechanisms include disruption of basal lamina from pericyte degeneration, release of pro-inflammatory factors from glial cells, reduced myelin repair from oligodendrocytes and reduced clearance capacity from neurons, to name a few. Each of these alterations can contribute to WM pathology seen in dementia syndromes.

### 2.2. Vascular heterogeneity across the normal brain and aging

#### 2.2.1. White matter has a poorer cerebrovascular reserve compared with gray matter

The cerebral vasculature is a continuum from arteries to veins which differ across the brain (Table 2) (39). GM regions are characterized by a greater number of large vessels and capillaries compared with WM (40), particularly in DGM regions, such as the hippocampus (41, 42). This has been well described in a cohort of 42 healthy young adults wherein vascular density for both arteries and veins was found to be lower in WM compared with GM, where it correlated with cortical thickness (43). Furthermore, GM and WM structures are morphologically heterogeneous, with energy supply being proportionally linked to the extent of connectivity between brain regions. The cerebrovascular reserve in GM is related to a rich neuronal, synaptic, and glia network demonstrating concomitant higher energy use, oxygen demand, and perfusion compared to WM (44). Therefore, differences in WM and GM vasculature, including changes to the structure and function of the NVU and BBB, and how they impact CBF should be kept in mind when considering studies on healthy aging and neurodegenerative disorders. Importantly, given the often-striking regional patterns of neurodegeneration seen in dementia syndromes, regional differences in these vascular structures and CBF warrant study.

**Table 2:** Heterogeneity of vascular tree [based on Schaeffer and Iadecola (39)].

Element of vascular tree	Vascular cells	Perivascular cells
Artery	Smooth muscle: Socs3, Atf3, Id2, Csrnp1 Endothelium: Gkn3, Hey1, Vcam, Vwf	Fibroblasts, mast cells, perivascular macrophages, extrinsic nerves
Arteriole (pial)	Smooth muscle: Slc26a2, Ttr, Sh3bgrl2, Tmem255b Endothelium: Gkn3, Hey1	Fibroblasts, mast cells, pial cells, perivascular macrophages
Arteriole (pre-capillary)		Astrocytes, microglia, intrinsic nerves
Capillary	Pericytes Pdgfrb, Cspg4, Vtn Endothelium Mfsd2a, Rgcc	Astrocytes, microglia, intrinsic nerves
Venule	Smooth muscle: Sebox, Tnxb, Ggt1, Grm3 Endothelium: Slc38a5, Lcn2	Astrocytes, microglia, intrinsic nerves
Vein	Smooth muscle: Sebox, Tnxb, Ggt1, Grm3 Endothelium: Slc38a5, Lcn2, Car4, Vwf, Vcam	Fibroblasts, mast cells, perivascular macrophages, extrinsic nerves

#### 2.2.2. Cerebral blood flow is physiologically reduced in white matter

Recent advances in neuroimaging support that CBF should not be considered as a global constant. Normal CBF is considered to be around 50 mL/100 g.min^−1^ in the healthy brain, and it is well established that CBF in WM is less compared to GM (~20 and 80 mL/100 g.min^−1^, respectively), reflecting the poorer cerebrovascular reserve described above (17, 45). This is a consistent observation across the brain (45), as well as in the cerebellum (46). It is also well established that stress and aging lead to reductions in CBF that are more severe in WM compared to GM (47). Interestingly, WM vulnerability is linked to the degree of network integration and connectivity, as demonstrated by a study looking at CBF and glucose metabolism (48). These findings support that CBF is influenced not only by intrinsic morphological differences in WM and GM structures, but also by loss of connectivity between them that can occur with aging.

#### 2.2.3. The blood–brain barrier is more vulnerable in periventricular WM with aging

Despite limited knowledge about region-specific BBB alterations in the healthy brain, a range of studies demonstrate vulnerability of WM and DGM to BBB and NVU damage with aging.

It is intuitive to postulate that molecular trafficking across the BBB is influenced by the distribution of the cerebrovasculature. It is well known that capillaries are more vulnerable (as the basal lamina is incomplete and pericyte coverage is lacking) in DGM (hippocampus, hypothalamus) and the surrounding WM, compared to CGM (49, 50). With aging, lower expression of TJ proteins has been reported in the periventricular WM (corpus callosum) compared with CGM, and morphological changes in ECs associate with BBB breakdown in WM but not GM following systemic inflammation (51). Prominent loss of pericytes has also been specifically observed in WM of pericyte-deficient mouse models, associating with accumulation of fibrinogen, a surrogate of BBB dysfunction (52). As fibrinogen is highly toxic for oligodendrocytes (53), fibrinogen accumulation may also play a major role in oligodendrocyte loss leading to WM changes and cognitive decline with aging (54). Furthermore, astrocyte morphology and distribution are highly heterogenous across the central nervous system (CNS), with elongated astrocytes with few processes in WM tracts (55), in comparison with highly branched protoplasmic astrocytes in GM (56). In mice, an age-dependent increase in GFAP astrocyte subpopulations, specifically in CGM and DGM structures (frontal, temporal cortices and hippocampus), has been shown (57). These observations highlight the role of aging on vascular dysfunction in WM and GM, which may set the stage for the evolution of cognitive impairment in susceptible individuals.

## 3. Vascular dysfunction in vascular dementia

The entity of vascular dementia and the pathologies encompassed by the term have posed challenges over the decades (58). However, vascular dementia is likely the second most common cause of dementia (the lack of certainty of this likely reflects historical disagreement over the definition of the condition), with mean autopsy prevalence rates of 8–15% in the West and higher in parts of Asia (59). The interactions between the vasculature, WM alteration and dementia were recognized as early as the nineteenth century, where arteriosclerosis, WM atrophy and preservation of CGM were associated with a slowly progressive dementia and gait disturbance (60). More recently, vascular cognitive impairment (VCI) has been defined as “a syndrome with evidence of clinical stroke or subclinical vascular brain injury and cognitive impairment affecting at least one cognitive domain” (61), and includes the entities of post-stroke dementia, multi-infarct dementia (due to multiple cortical infarcts) and subcortical ischemic vascular dementia (SIVD) (58). In particular, the dementia associated with small vessel disease (SVD) pathology of the WM (SIVD) is characterized by a frontal dysexecutive syndrome, slowed processing speed and sustained attentional deficits (62). Another important entity linking vascular pathology and dementia is cerebral amyloid angiopathy (CAA).

**Figure 2:**
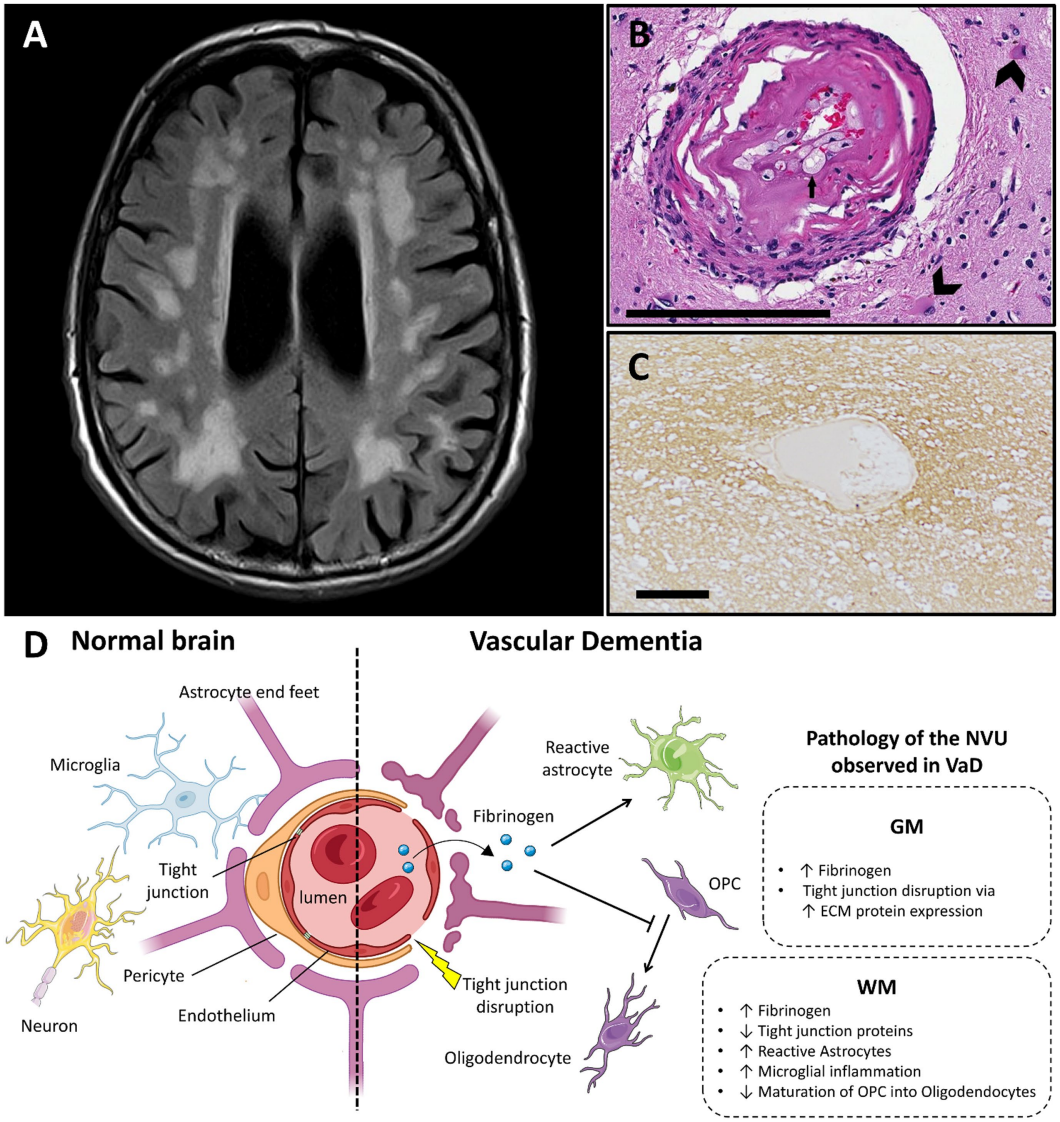
Vascular dementia and vascular dysfunction. **(A)** Typical MRI features of small vessel disease, showing subcortical and periventricular white matter hyperintensities (case courtesy of Frank Gaillard, Radiopaedia.org, RID: 25641). **(B)** Arteriole showing hyalinization of the vessel wall. Small arrow highlights a foamy macrophage in the hyalinized part. Large arrows show astrocytes in the surrounding neuropil (adapted from (74) with permission; H&E stain; scale bar 200 μm). **(C)** Fibrinogen deposition in a perivascular distribution in a white matter lesion in small vessel disease, shown with immunohistochemistry (adapted from (83) with permission; scale bar 50 μm). **(D)** Main features of blood–brain barrier and neurovascular unit alterations in vascular dementia compared with healthy brain (created with BioRender.com; OPC, oligodendrocyte progenitor cells; GM, gray matter; WM, white matter; ECM, extracellular matrix; VaD, vascular dementia; NVU, neurovascular unit).

In the next section, we explore the regional specific perfusion alterations and neuropathology of SVD (Figure 2), including insights from the rare monogenic subcortical arteriopathy syndromes associated with dementia, such as Cerebral Autosomal Dominant Arteriopathy with Subcortical Infarcts and Leukoencephalopathy (CADASIL).

### 3.1. Hypoperfusion in vascular dementia

By definition, chronic hypoperfusion is central to VCI, wherein a 20–35% reduction in global CBF has been observed (63). This is supported by metanalyses which demonstrate a relationship between severity of WM hyperintensities (WMH, the cardinal manifestation of SIVD) and reduced CBF (64, 65). In aged patients with dementia, regions with WMH had lower CBF than normal appearing white matter (NAWM) and periventricular WM had lower CBF than other WM areas (66). It was also shown that low baseline CBF in normal appearing periventricular WM can predict the progression of WMH in aged patients.

Interestingly, changes to CBF extend beyond WM, and also involve CGM. Regional differences in CBF and their relationship to WMH were studied in a cohort of MCI patients wherein VCI is commonly observed. A relationship between reduced CBF and WMH was seen in parietal, occipital and temporal CGM but not frontal CGM; these findings were not accompanied by overlying cortical atrophy implying an early role for vascular dysfunction (67). In another study, GM atrophy was observed in SIVD, in particular in prefrontal cortex, the middle and superior temporal gyri and the thalamus (68), although this study did not look at CBF. The relationship between CBF alterations and patterns of atrophy therefore warrant further study.

Taken together, these studies highlight that regional CBF changes, even in GM, are associated with WM lesions seen in SIVD, detectable even in early disease phases. What is known about the pathological substrate of these CBF changes in vascular dementia will be discussed below.

### 3.2. Neuropathology of vascular dysfunction in vascular dementia

Pathologically, SIVD is characterized by hyalinization of the vessel walls, fibrinoid necrosis, widening of the perivascular spaces, perivascular demyelination, tissue ischemia, and astrogliosis (69, 70). These features are influenced by hypertension, a major risk factor for SIVD. As WM is inherently vulnerable to cerebrovascular insults (71, 72), it is not surprising that prior studies have shown that the extent of subcortical WM vascular disease correlates with the presence of dementia (69, 73). Of important note, these changes in the small vessels do not occur in isolation, and have been associated with atherosclerotic disease of the larger cerebral vessels (74).

#### 3.2.1. The blood–brain-barrier in vascular dementia

BBB permeability has been described in SVD but the results have been conflicting. Although one study reported that surrogates of BBB permeability, such as fibrinogen and IgG, in DGM and subcortical WM do not correlate with pathological measures of SVD, nor with MRI measures of leukoaraiosis in life (75), many studies have found evidence of BBB breakdown. In fact, BBB breakdown has been described in the perivascular space of DGM in SVD cases (76). A spectrum of animal and post-mortem studies support BBB dysfunction in SVD, wherein hypertension damages ECs of small cerebral vessels (77), and associates with increased fibrinogen and IgG deposition (DGM and WM) in the brain, particularly in astrocytes located in periventricular WM (78).

In addition to accumulation of toxic factors, ECs dysfunction is evidenced by reduced TJ protein expression in both rat models of SVD and deep WM of people with early stage SVD (79). Reduced endothelial nitric oxide synthase (eNOS), which results in vasoconstriction and ultimately tissue ischemia (80), is another marker of EC dysfunction that has been described in SVD. Decreased eNOS exacerbates BBB leakage, WM damage and dementia in animal models (81). Dysfunctional ECs can also secrete ECM-related proteins which disrupt TJs in DGM of animal models (82). As a consequence, BBB breakdown can lead to changes in the NVU promoting tissue damage, in both WM and GM, mirroring CBF changes detected in imaging studies.

##### 3.2.1.1. The neurovascular unit in vascular dementia

Although vascular pathology is thought to be the underlying mechanism for leukoaraiosis, glial dysregulation is also of interest (6). Components of the NVU may be compromised early in SIVD, including astrocytes, microglia and oligodendrocytes.

Increased reactive astrocytes are seen in WM lesions in aged patients, and are positive for fibrinogen accumulation, indicating BBB breakdown (78). Of note, the astrocytic network pattern found in SIVD is similar to that observed in MS WM demyelinated lesions. In mouse models of cerebral hypoperfusion, blockade of astrogliosis reduces demyelination and cognitive impairment (84).

Altering microglial activity can influence WM pathology. For example, microglial polarization to a less inflammatory phenotype (via fingolimod in mice) leads to preservation of both WM integrity and cognitive function (85, 86). Similarly, pioglitazone, a peroxisome proliferator-activated receptor γ agonist, ameliorates WM lesion scores and performance on the Morris water maze in stroke-prone hypertensive rats; these findings were associated with reduced proliferation of astrocytes and microglia with reduction of pro-inflammatory cytokines (87).

In rat models and human tissue of early SVD, increased numbers of oligodendrocyte progenitor cells (OPCs) have been detected and attributed to reduced maturation into oligodendrocytes (78); interestingly, drug treatment targeting EC dysfunction was found to promote OPC differentiation into oligodendrocytes (79). This is further supported by the observation that fibrinogen extravasation across a disrupted BBB and ischemia-induced oxidative stress impede OPC differentiation and maturation (6). The conflation of these findings highlights the impact of the NVU on glial function and WM integrity relevant to cognitive function.

##### 3.2.1.2. Insights from hereditary microangiopathies

Several genetic diseases of the small cerebral vessels are associated with dementia. These include CADASIL (due to mutations in the *NOTCH3* gene) and CARASIL (cerebral autosomal recessive arteriopathy with subcortical infarcts and leukoencephalopathy, due to mutations in the *HTRA1* gene) (74). These diseases may inform pathological processes involved in the much more common sporadic SIVD. *NOTCH3* encodes for a transmembrane receptor expressed in pericytes and vessel smooth muscle cells and is important for their integrity. There are many identified mutations within the gene and the disease often arises from deposition of abnormal NOTCH3 protein within the vessel walls, resulting in changes similar to that seen in SIVD (88). *HTRA1* (high temperature requirement a serine peptidase), mutated in CARASIL, causes loss of repression of TGF-beta, a growth factor, with resultant vascular fibrosis. A similar pathology is thus seen in CARASIL and these patients develop dementia earlier than in CADASIL, as the changes are more widespread (89). Therefore, both these conditions act as informative models of SIVD, in that they result in arteriole wall hyperplasia and resultant reduced blood flow and ischemia (89). Indeed, many of the imaging studies on CBF in cerebrovascular disease were done on cohorts of patients with CADASIL (90).

## 4. Vascular dysfunction in multiple sclerosis

**Figure 3:**
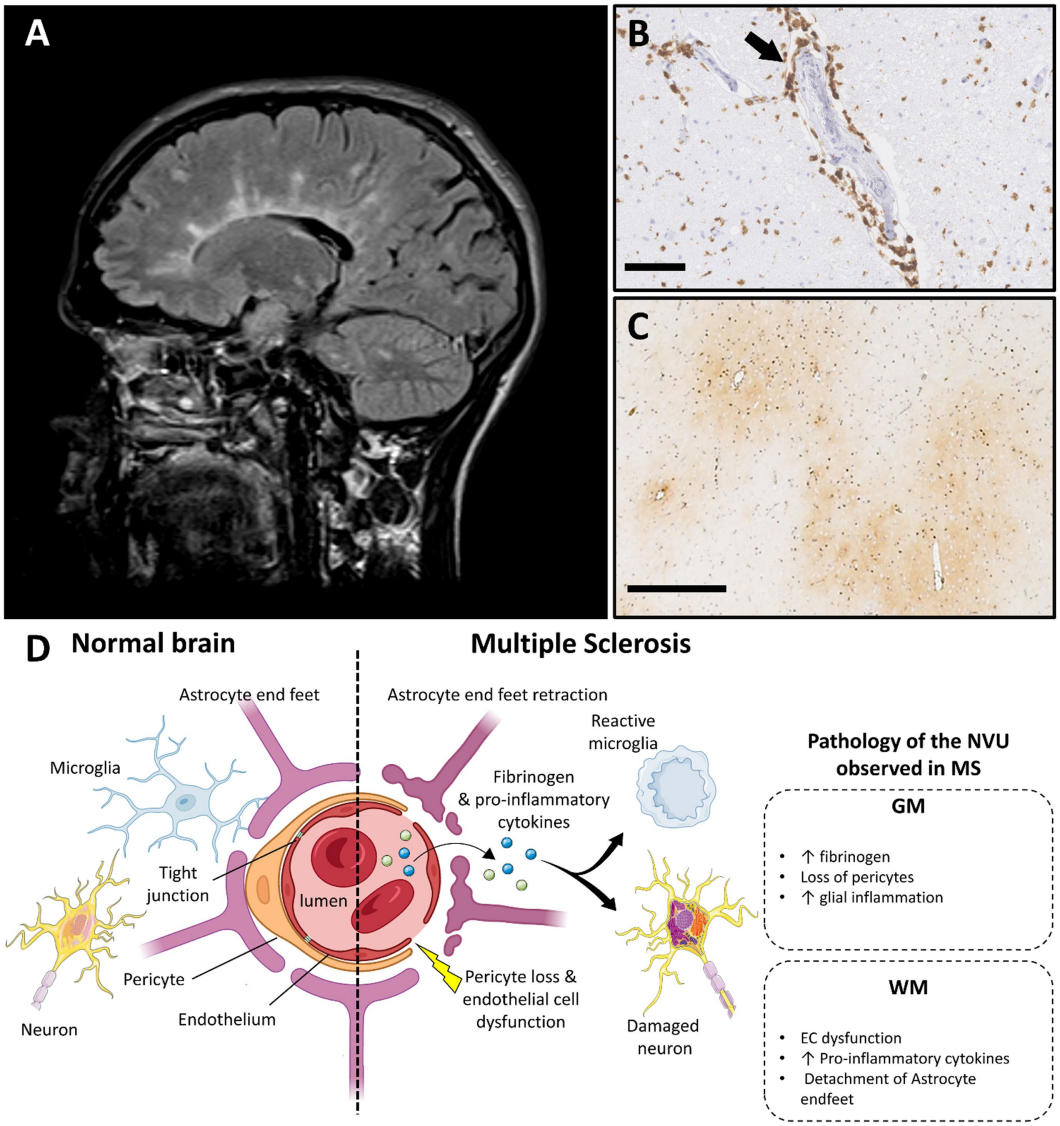
Multiple sclerosis and vascular dysfunction. **(A)** Periventricular white matter lesions in a typical Dawson’s finger distribution in a person with multiple sclerosis on T2 weighted MRI image (FLAIR; case courtesy of Frank Gaillard, Radiopaedia.org, RID: 1067). **(B)** Perivascular lymphocytic inflammation in an acute MS plaque, immunohistochemistry for cluster differentiation 3 (CD3) (scale bar 100 μm). **(C)** Perivascular fibrinogen deposition is demonstrated with immunohistochemistry in the primary motor cortex (frontal cortex), in non-demyelinated gray matter area (figure adapted from before renumber no (138) with permission; scale bar 500 μm). **(D)** Main features of blood–brain barrier and neurovascular unit alterations in multiple sclerosis compared with healthy brain (created with BioRender.com; GM, gray matter; WM, white matter; EC, endothelial cells; MS, multiple sclerosis; NVU, neurovascular unit).

MS is an inflammatory disorder of the CNS estimated to affect more than 2.8 million people worldwide (91). MS is characterized by inflammation, demyelination, and neurodegeneration that results in disability and reduction of quality of life (92). Changes to cognition and emotion are common features, an observation which dates back to Jean-Martin Charcot’s classic descriptions (93). More recent studies suggest that cognitive impairment is present in 34% of those with clinically isolated syndrome (CIS), 50% with relapsing–remitting MS (RRMS), and 80–90% of those with progressive MS (94). Interestingly, the cognitive impairment seen in MS has several similarities to that seen in vascular dementia, namely dysexecutive symptoms, slowed processing speed, and impaired sustained attention (95). Further, it is increasingly recognized that GM alterations may be associated with vascular disturbances in MS (Figure 3) and likely play an important role in cognitive decline. Indeed, GM demyelination is frequent and extensive in MS, both in CGM and DGM structures relevant to cognitive function (e.g., thalamus, hippocampus, cerebellum) (96–98). Vascular dysfunction is also increasingly recognized as an early feature of MS pathogenesis, supported by the pathognomonic vessel-associated pathology characteristic of the disease (99).

We herein provide a summary of the main characteristics of vascular dysfunction in MS, including its regional variability.

### 4.1. Perfusion alterations in multiple sclerosis

The clinical pattern of MS dementia resembles that of vascular dementia with similarities in CBF hypoperfusion patterns being seen. While the formation of a new demyelinating plaque is accompanied by an increase in CBF, the MS brain gradually displays progressive reduction in CBF which correlates with the degree of atrophy and clinical disability.

Hyperperfusion has been shown to be one of the earliest events in plaque formation detectable on MRI, being present even before emergence of gadolinium-contrast enhancement and changes in diffusion (100). This relative hyperperfusion (18% increase from baseline) in active WM lesions has been attributed to vasodilation in response to an intense inflammatory response (101). This notion is supported by studies evaluating the experimental autoimmune encephalomyelitis (EAE) mouse model of MS, wherein induction of severe inflammation results in severe hypoxia (102), and consequent increased perfusion. Interestingly, oxygen administration not only attenuated hypoxia but also clinical deficits (103).

Outside the acute inflammatory phase of plaque formation, the available MRI literature consistently reports hypoperfusion across brain regions in all disease stages of MS, wherein a 20% reduction in global CBF has been reported in people with MS compared with normal aging (104). In fact, normal appearing periventricular WM and DGM and CGM areas are particularly affected (105–108) with CBF reduction of up to 50% being reported. The reduced global CBF has clinical relevance as it correlates with measures of brain atrophy, and cognitive and motoric disability (109–111), independent of vascular risk factors (109). In early disease stages, CBF hypoperfusion has been shown to preferentially occur in juxtacortical and periventricular WM areas and associate with severe demyelination with relatively sparing of CBF alterations in CGM and DGM areas. It has been suggested that secondary hypoperfusion in GM areas due to underlying WM injury may contribute to disease progression in later disease stages (112, 113) but this warrants further study. Regardless, the observation that CBF hypoperfusion occurs at the earliest disease stages in normal appearing WM areas further supports the notion that CBF alterations are not merely a downstream consequence of MS pathology but rather may be an important upstream contributor of it (112).

### 4.2. Neuropathology of vascular dysfunction in multiple sclerosis

Vessel-associated inflammatory demyelination is a hallmark of MS pathology, especially in WM regions. In recent years, a growing body of evidence suggests that vascular changes occur outside of WM lesions and affect the vascular tree beyond the traditionally blamed venule. The observation that vascular risk factors, which are known to impact the arterial system, associate with more severe pathology and disease progression supports this claim (114). A recent post-mortem study showed that peri-arterial small vessel disease outside of lesions is increased and associates with inflammatory disease activity in progressive MS (99).

#### 4.2.1. Pathology of the BBB and NVU in demyelinated plaques

In MS, WM lesion burden associates with the development of cognitive deficits thereby pointing to a role for vascular dysfunction in cognitive decline (115–117). MRI studies have highlighted the importance of BBB alterations in the pathogenesis of WM pathology as evidenced by the presence of gadolinium enhancement in acute lesions. BBB breakdown occurs at the earliest disease stages and associates with extensive perivascular immune cell infiltration in WM lesions (118–125). Dysfunctional ECs have been shown to propagate the inflammatory response in WM lesions by upregulating membrane receptors (e.g., TLR4) (126, 127) and inducing release of proinflammatory cytokines (e.g., IFN-γ and TNF-α) by glial cells; these changes, in turn degrade TJs and surrounding ECM to form a vicious circle of BBB breakdown (118, 128, 129). BBB integrity is further compromised in active WM lesions by the detachment of astrocyte endfeet from the basal lamina (130). Further support for a role for astrocytes in WM lesion pathogenesis is derived from the finding of increased astrocyte-derived vasoconstrictive peptide endothelin-1 (ET-1, related to reduced CBF) in CSF of MS patients (131). The observations support early changes to BBB integrity in the pathogenesis of WM lesions.

Surprisingly, little is known about vascular dysfunction in GM lesions, despite extensive involvement of GM pathology in MS. In contrast to WM lesions, some studies report no alterations in ECs and their TJs, as well as no differences in infiltrating lymphocyte numbers in cerebral cortical lesions compared to NAGM (132). These findings are challenged by recent neuroimaging and post-mortem studies that show loss of BBB integrity in CGM and WM lesions alike and extensive inflammatory activity in hippocampal and spinal cord GM lesions, respectively (133–137). BBB disruption in both GM and WM areas are likely substrates, which highlights the need to consider the role of vascular dysfunction in these areas and how they contribute to cognitive impairment in MS.

#### 4.2.2. Pathology of the BBB and NVU beyond demyelination

The classical view of BBB disruption as only a feature of demyelinated lesions in MS is challenged by a body of evidence that implicates BBB breakdown in NAWM and NAGM. Extensive fibrinogen accumulation, a surrogate of BBB disruption, has been detected in NAGM areas in progressive MS; the fact that the extent of fibrinogen deposition correlates with neuronal loss, the substrate of irreversible disability, adds clinical relevance to this finding (138, 139). The mechanism by which fibrinogen, a large molecular weight protein, traverses the BBB has attracted studies evaluating the role of various constituents of the BBB in fibrinogen egress from the vasculature. A mouse model of pericyte-deficiency showing accumulation of parenchymal fibrin supports involvement of pericyte dysfunction in this process (138, 140). In support of this possibility, EAE rat models demonstrate pericyte loss in CGM capillaries with induction of PDGFRβ expression associating with overexpression of TJs proteins (claudin) and reduction of clinical symptoms (141). Astrocyte activation and alterations of aquaporin-4 expression in their end feet have been also shown to contribute to vascular alterations even before the infiltration of immune cells (142, 143). The accumulation of fibrinogen in astrocyte cell bodies and processes in the glia limitans and perivascular areas of non-lesional areas further implicate a role of astrocyte function and fibrinogen accumulation (138, 139). Importantly, fibrinogen is known to initiate and propagate neurotoxic microglial activation in EAE mouse models leading to the secretion of pro-inflammatory cytokines (e.g., TNF-α, IL-1β) that contributes to neurodegeneration (144, 145). This is supported by recent neuropathological findings in NAGM cerebral cortical areas suggesting that neurotoxic factors, opposing putative microglial-associated protective factors, are secreted by fibrinogen-stimulated microglia in people with HLA-DRB1*15 genetic status, a major risk factor for MS, further highlighting a pathologically relevant consequence of BBB compromise in the disease (146). Future work evaluating the nature and extent of BBB and NVU changes in both WM and GM within and outside lesions throughout the MS brain will help disentangle their roles in disease pathogenesis.

Overall, these findings demonstrate that changes to the BBB and NVU are important features of MS pathology even beyond areas of demyelination in both GM and WM and contribute to neurodegenerative processes relevant to cognitive impairment in MS (99).

## 5. Alzheimer disease and vascular dysfunction

**Figure 4:**
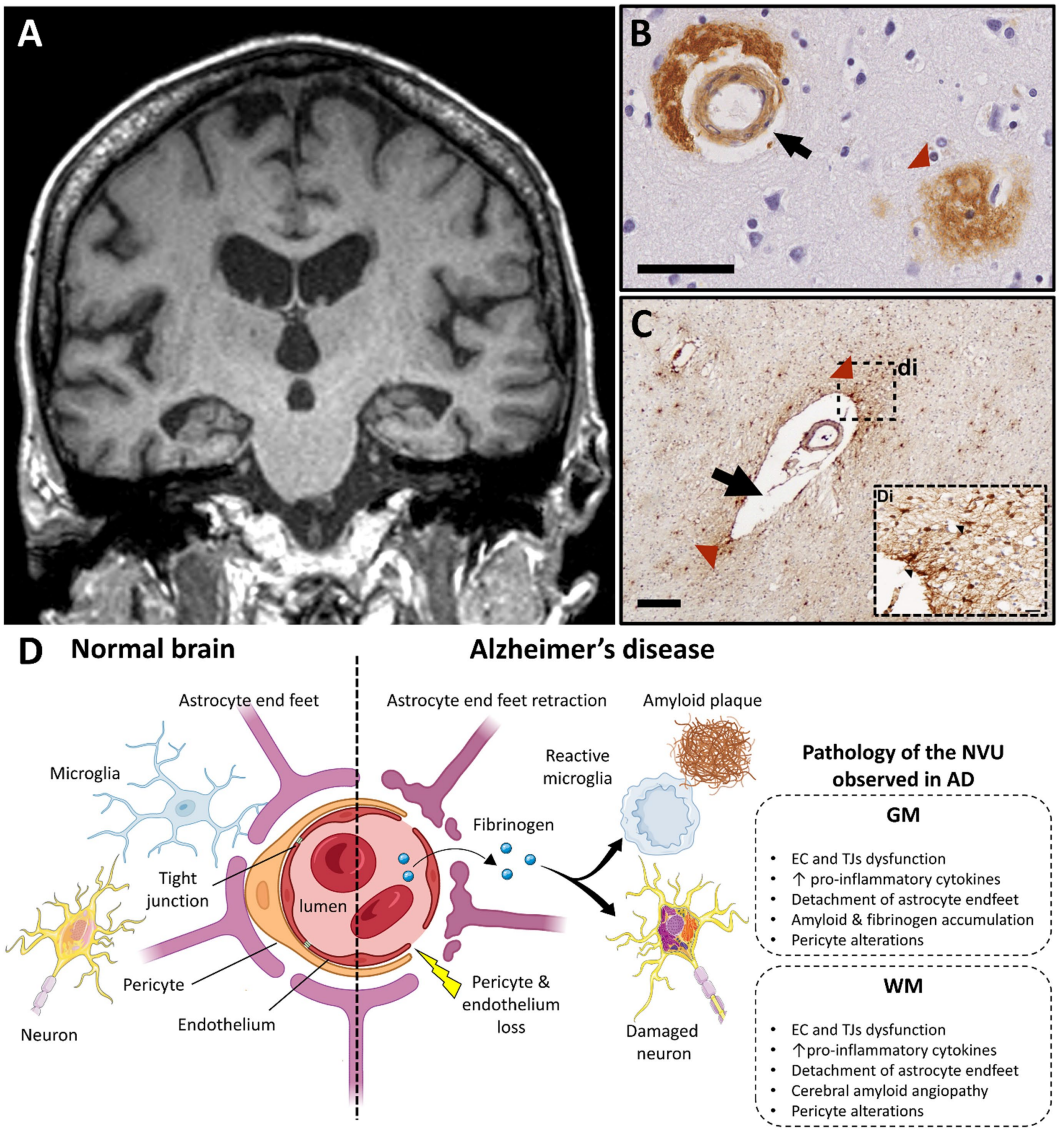
Alzheimer’s disease and vascular dysfunction. **(A)** Severe and bilateral atrophy of hippocampi is a typical MRI characteristic of Alzheimer disease (T1 weighted brain MRI scan; case courtesy of Frank Gaillard, Radiopaedia.org; RID: 22196). **(B)** Immunohistochemistry for β-amyloid demonstrates cerebral amyloid angiopathy in the vessel wall of capillaries (black arrow) and in an amyloid plaque, which are both typical features of AD pathology (scale bar 50 μm). **(C)** A vessel with a dilated perivascular space (black arrow) and perivascular fibrinogen deposition (red arrowhead) is demonstrated with immunohistochemistry in the deep white matter in the parietal lobe; (Di) Magnified insert show extravasation of fibrinogen in the white matter parenchyma (black arrowheads, figure adapted from McAleese et al., 2019 with permission; scale bar 200 μm). **(D)** Main features of blood–brain barrier and neurovascular unit alterations in Alzheimer’s disease compared with healthy brain (created with BioRender.com; GM, gray matter; WM, white matter; EC, endothelial cells; TJ, tight junction; AD, Alzheimer’s disease; NVU, neurovascular unit).

AD is a neurodegenerative syndrome characterized by early deficits in working and episodic memory with an increased incidence and prevalence as the population ages. There are in excess of 55 million people living with dementia worldwide and 60–70% of these have AD (147). AD is characterized by brain atrophy, neuroaxonal loss and accumulation of amyloid-β and Tau, mainly in GM structures. However, recent studies also highlight significant WM changes in AD pathology. Regional vascular abnormalities are likely important contributors to both GM and WM pathology in AD (Figure 4) with some features shared with vascular dementia and MS.

### 5.1. Cerebral blood flow impairment in Alzheimer’s disease

Compared to normal aging, reduction of 20–40% of the global CBF is common in AD (148). In particular, it is well-established that CGM and DGM hypoperfusion, mainly in the temporal lobe, are early features of the disease. In MCI and early stages of AD, a wide range of studies show reduced CBF in DGM (such as hippocampus, thalamus and basal ganglia) (149–154) and CGM (temporal cortex) (155, 156), which often associate with progressive cognitive decline and brain atrophy. Relative hyperperfusion has also been described in early AD, which has been attributed to a transient compensatory response to local inflammatory and neurodegenerative mechanisms (157–160). With AD progression, hypoperfusion in CGM areas (estimated to be about 20 mL/100 g.min^−1^ by MRI) (161), such as frontal and parietal cortices, relates to hypometabolism, amyloid accumulation,and cognitive decline (162–165). The extent to which CBF alterations drive or are a consequence of AD pathology requires further study.

In addition to GM, changes to CBF in WM have been described, especially at more advanced disease stages when subcortical cognitive change is a more prevalent feature. In established AD, up to 50% reduction of CBF in temporal, parietal and occipital WM areas has been shown to be more severe than in their GM counterparts (up to 40% reduction) (166, 167). These CBF changes relate to WM hyperintensities (WMH) often observed in AD (25), which variably associate with cognitive impairment (168, 169), especially when situated in periventricular and juxtaventricular areas (170). Hypoperfusion in the corpus callosum has been described in late, but not prodromal, stages of AD highlighting an important temporal element to WM involvement in AD pathogenesis (159, 171, 172). In fact, such stage-dependent regional CBF alterations in WM in AD are hypothesized to disrupt WM integrity and network connectivity between GM regions, further exacerbating cognitive decline (173–175). Studies of the brain connectome will shed further light into the relative roles of CBF changes in WM and GM at various stages of AD-related cognitive decline.

### 5.2. Neuropathology of vascular dysfunction in Alzheimer’s disease

To date, the unequivocal diagnosis of AD relies on post-mortem detection of amyloid and Tau in characteristic brain regions. However, amyloid and tau deposition do not fully account for the progressive cognitive decline seen in AD, implying that other factors, such as vascular pathology, are contributing to AD pathology. Reports of either unchanged, reduced or increased vascular densities in the AD brain complicate interpretation of vascular changes in AD pathogenesis, although differences in methods, AD severity and selected controls may explain these discrepant findings. However, there is increasing recognition that loss of connectivity between WM and GM regions and vascular dysfunction are contributors to cognitive decline and may augment other aspects of AD pathology. In that context, more is known in AD than any other dementia, wherein it is suggested that vascular abnormalities affect both WM and GM through the “two-hit vascular hypothesis” (176, 177). The first hit is damage to the microcirculation independent of changes to amyloid burden, while the second hit is loss of ECs and TJs supplemented by NVU alterations that lead to impaired Aβ clearance and subsequent amyloid accumulation in the AD brain.

#### 5.2.1. Evidence of white matter pathology in Alzheimer’s disease

An increasing body of evidence suggests that WM changes play an important role in AD pathophysiology, including WM degeneration and demyelination secondary to reduced numbers and function of OPCs and oligodendrocytes, disrupted connectivity of WM tracts, and amyloid-induced inflammatory damage (178–182). The finding that *ApoE4* carriers show significant WM degeneration prior to emergence of GM atrophy and cognitive dysfunction further highlights a fundamental role of WM in AD disease pathogenesis (183). Advanced MR studies show altered vascular integrity in WM areas adding further support to a role vessel-related WM changes in AD (176, 184).

Another key aspect of vascular change linked to WM damage in AD is cerebral amyloid angiopathy (CAA). CAA is characterized by the accumulation of amyloid in the wall of the cerebral vessels and occurs in up to 90% of established AD cases (185). CAA is associated with prominent ischemic WM damage and atrophy (186, 187) secondary to extravasation of immune cells and toxic factors. The accumulation of fibrinogen in neurons and vessels in areas of Aβ deposition (188) and the formation of Aβ-fibrinogen insoluble clots in the parenchyma propagate deleterious inflammatory responses, vascular dysfunction, and CAA that contribute to WM change in AD (189, 190).

#### 5.2.2. Pathology of the blood–brain barrier in Alzheimer’s disease

Macroscopic vascular changes reflect BBB alterations observed in AD. Increased numbers of fragmented vessels and branches (191, 192), vessel tortuosity as well as the presence of non-functional and degenerative capillaries are commonly observed in AD (191, 193). Intracranial atherosclerosis, especially in the hippocampus and CGM areas, mirrors the distribution of the aforementioned CBF changes (194, 195). While atherosclerosis in these eloquent areas associates with cognitive decline in AD (196), concomitant CBF alterations in subcortical WM areas and corpus callosum during disease progression may be also to blame.

In AD, BBB breakdown contributes to neurodegeneration, amyloid accumulation and neuroinflammation (71). Loss and/or degeneration of TJs, EC and pericytes and accumulation of toxic factors, such as fibrinogen and immunoglobulins (IgG), are features of BBB disruption in AD (197–202). Breakdown of the BBB in the hippocampus has been shown to precede behavioral deficits and cognitive impairment in AD animal models (203, 204) and AD patients (205), respectively, with alterations to endothelial TJs being independent of amyloid burden. Similar features are seen in periventricular WM, where shortened TJs are commonly observed (191, 206). In addition, thickening of the vascular basal lamina is thought to play an important role in AD-related neurovascular changes with alterations of vessel-associated ECM proteins being a culprit (207, 208). Other ECs alterations are postulated to be central players in AD pathogenesis, which are reviewed in detail elsewhere (209).

NVU alterations mirror the regional and temporal evolution of CBF and BBB changes seen in AD. Temporal lobe pathology is a consistent, early feature with involvement of other cortical GM and subcortical WM regions being seen in later AD stages. In a model of adult viable pericyte-deficient mice, BBB breakdown and neuronal loss occurred in both GM and WM structures in an age-dependent fashion (140). In early AD, pericytes accumulate Aβ in order to degrade it, while Aβ oligomers reduce CBF through pericyte-mediated capillary constriction, forming a vicious circle of dysfunctional clearance and energy supply (210). Pericyte alterations associate with BBB breakdown before extensive amyloid deposition in the AD hippocampus (205), and are detected in later stages and in other cortical regions when amyloid burden is severe (211, 212). Pericytes are also affected in subcortical WM during AD progression (9, 52, 213) Further studies evaluating the nature, timing, and extent of pericyte responses to AD-relevant stimuli and how they impact the NVU in GM and WM are needed.

Activation of perivascular glial cells, such as astrocytes and microglia, are thought to be secondary features of BBB breakdown and pericyte alterations in AD. Activation of perivascular astrocytes occurs in the vicinity of amyloid plaques and leads to propagation of local neuroinflammation in CGM and DGM structures in established AD (214–218). Interestingly, a specific subtype of tau-positive perivascular astrocytes in the subcortical WM associates with brain atrophy (219, 220). Microglial activation and dysfunction are linked to oxidative stress that exacerbates neurodegeneration in CGM areas, such as frontal, temporal and cingulate cortices, as well as underlying WM in AD (221, 222). In addition, microglia are involved in vascular remodeling. Microglia amplify and sustain local inflammation in response to fibrinogen accumulation in the AD entorhinal cortex (223). This is further supported by fibrinogen-mediated microglial activation in the spinal cord of an AD mouse model which exacerbates neurodegeneration and subsequent cognitive decline, independent of amyloid accumulation (224). These findings support an important role for glial activation and dysfunction in AD pathogenesis that requires further study.

#### 5.2.3. Aβ accumulation impacts blood–brain barrier integrity

BBB dysfunction in CGM and DGM contributes to impaired Aβ clearance in the AD brain. Aβ can reduce EC-related LRP1 activity, a protein known to regulate the clearance of several toxic factors including Aβ itself (225, 226). Fibrillar Aβ has been reported to induce pericyte loss and apoptosis in the AD hippocampus (227), while oligomeric Aβ induces pericyte-mediated hypoperfusion in CGM (parietal cortex) (212). Aβ is known to interact with the receptor for advanced glycation end-products (RAGE), which mediates the accumulation of Aβ from ECs to neurons leading to the release of proinflammatory factors and neuronal loss (225, 228, 229). In addition, ECs derived from rat cortices subjected to Aβ treatment show alterations in TJs through impaired Zo-1 and occludin expression (230). Importantly, the contribution of amyloids to vascular dysfunction is highlighted by the emergence of amyloid-related imaging abnormalities (ARIA) in AD patients treated with novel amyloid-lowering monoclonal antibodies. ARIA is thought to relate to BBB disruption secondary to the mobilization of amyloids previously deposited in blood vessels with oedema and hemorrhage subtypes being described (231, 232). Altogether, these findings provide evidence that Aβ can induce BBB alterations that not only reduce its own clearance but also augment subsequent neuroinflammation and neurodegeneration, mainly in CGM and DGM areas. Very little is known about the relationship between the (marginal) amyloid deposition in WM parenchyma and vascular dysfunction and should be the focus of future study.

## 6. The shared landscape of vascular dysfunction in WM dementia

We have discussed three diseases largely considered under separate sub-specialities of neurology: a vascular disorder (vascular dementia), an immune-mediated disorder (MS) and a neurodegenerative disorder (AD). Cognitive impairment is a unifying feature of these diseases with important similarities and differences between them. SIVD and MS both show early deficits in executive function, processing speed and attention classically termed “subcortical dementia” due to their predominant WM involvement. In contrast, AD shows early deficits in episodic memory, language and visuospatial processing consistent with what is termed a “cortical dementia” with predominant GM involvement. However, all three conditions have important WM and GM contributions at various disease stages with changes to the vasculature being a unifying culprit as highlighted in this review.

A central role for the vasculature in the pathogenesis of vascular dementia, MS, and AD is further supported by the influence of vascular risk factors upon their incidence and severity. Smoking, in particular, is an established risk factor for vascular dementia, MS and AD (233, 234). Mid-life vascular risk factors are predictive of dementia later in life (235). Whether these risk factors for vascular dysfunction are an early primary triggering step for pathological processes or whether they reduce the threshold for separate pathological processes to manifest clinically is unknown. However, the concept of one pathology triggering an accelerated manifestation of another has recently been observed in COVID-19, where infection was associated with molecular signatures of aging in the human brain (236).

In addition, genetic and “non-vascular” environmental influences are reported in WM dementias. Their impact on vascular dysfunction is poorly understood, and warrants further investigation, as it may shed light on pathological processes common to these diseases. People carrying the *ApoE4* allele are at high risk of AD and have alterations in BBB function (201), and it was recently shown that *ApoE4* may predict cognitive decline in CIS and early RRMS patients (237), however not directly linked to vascular dysfunction. Furthermore, environmental factors such as hypovitaminosis D and obesity, can influence WM dementias in terms of incidence and severity. As an example, hypovitaminosis D is a risk factor in vascular dementia, MS and AD (238–240). Interestingly, in animal models (not of the diseases discussed in this review), hypovitaminosis D affects CBF, BBB and NVU (241, 242). This finding should be explored further in the context of dementia.

**Figure 5:**
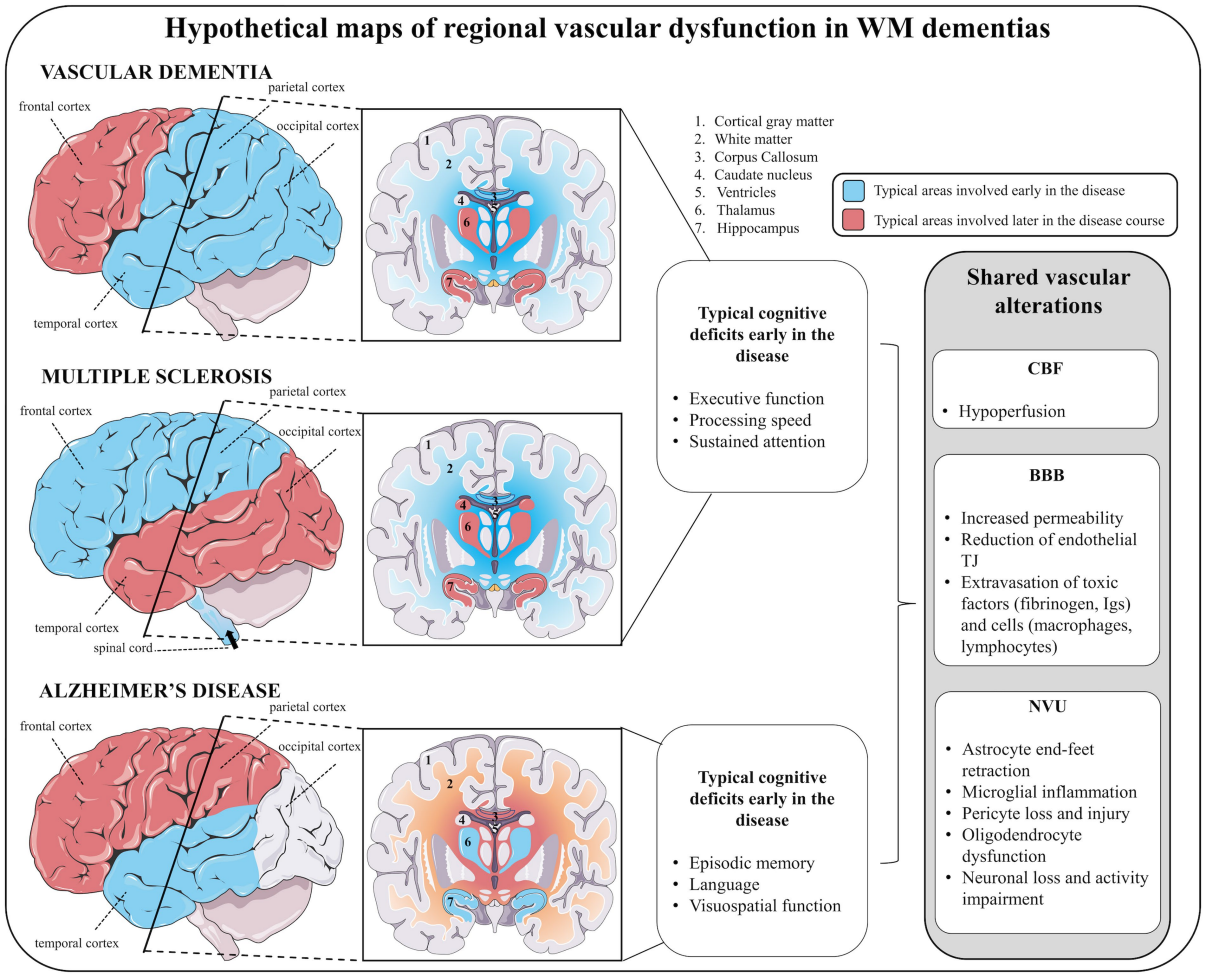
The shared landscape of vascular dysfunction in WM dementia. Hypothetical maps of regional vascular dysfunction in white matter dementias share similar cerebral blood flow and blood–brain barrier alterations. In vascular dementia, early vascular dysfunction is seen in temporal, parietal and occipital cortices, subcortical white matter (in particular the corpus callosum) followed by frontal cortex, and deep gray matter structures (hippocampus, hypothalamus). Similar to vascular dementia, in multiple sclerosis, early vascular dysfunction in parietal cortex (but also in frontal cortex), white matter (in particular the corpus callosum) followed by temporo-occipital cortices and deep gray matter structures (hippocampus, thalamus, and caudate nucleus) are features. In addition, vascular dementia and multiple sclerosis share similar early cognitive deficits, including impaired executive function, processing speed and attention. In Alzheimer’s disease, early vascular dysfunction is commonly encountered in the temporal lobe (including cortical and deep gray matter structures), followed by subcortical white matter (in particular the corpus callosum) and frontoparietal cortices. Early cognitive deficits in Alzheimer’s disease affect episodic memory, language and visuospatial processing. For each of these diseases, vascular disturbances are suggested to be linked to juxtaventricular areas, where WM damage is consistently observed.

The complexity of regional neuroinflammatory and neurodegenerative processes during progression (Figure 5) and the heterogeneity within and between the different WM dementias challenge our understanding of these diseases. Further, once the disease is established, the relationships between WM and GM degeneration can be distinct (243). The classical view of the regional differences in the diseases discussed in this work is that CGM and DGM damage are the drivers of AD pathology, while WM damage is the predominant process from early onset to late stages in MS and vascular dementia pathology. This review discusses a shared and fundamental role of vascular dysfunction in WM dementia which challenges such dichotomic view (Figure 5), wherein potential compensatory mechanisms early in these diseases should be explored. This is particularly true in vascular dementia, which occurs alone or as mixed dementia with comorbid AD (244). It is therefore important to consider the potential causal factors of such alterations, wherein a large spectrum of evidence points toward accelerated aging. Whether it concerns hypoperfusion, BBB permeability or dysfunctional NVU, all these features are observed in normal aging, which are exacerbated in the context of WM dementia and must be taken into account when trying to understand these diseases independently or jointly.

Despite a growing number of neuroimaging techniques to quantify WM and GM vascular properties in human brain, little is known regarding the spatial organization, the connectivity variations, and the distribution of the cerebral vasculature in different conditions, due to their relatively new development. It is complicated by the heterogeneity of inter-subject venous and arterial distributions wherein the regulation of molecule trafficking by the BBB varies in consequence. However, the emergence of new tools to quantify longitudinally vascular changes in neurodegenerative disorders, may provide an atlas of the human cerebrovascular system, which is of crucial relevance (43).

The unifying concept in WM dementia associated with vascular dementia, MS and AD, appears to lie within the fabric of the blood supply against the backdrop of an aging brain. Though WM pathology may not be the primary cause for cognitive decline, it plays an important role. The insights from the imaging and pathology studies discussed in this review can form the basis of an important public health message; identification and treatment of traditional vascular risk factors in middle age has the potential to modify pathological processes in the brain later in life across a much wider spectrum of neurological diseases than perhaps is widely recognized.

## Author contributions

GD conceived the concept of the review. JP, GH, and AL performed the literature review. JP, GH, AL, MP, and GD drafted the manuscript and edited for intellectual content. All authors approved the manuscript for submission.

## Funding

This work was supported by the UK-MS Society (HMR02630 and HMR04500).

## Conflict of interest

The authors declare that the research was conducted in the absence of any commercial or financial relationships that could be construed as a potential conflict of interest.

## Publisher’s note

All claims expressed in this article are solely those of the authors and do not necessarily represent those of their affiliated organizations, or those of the publisher, the editors and the reviewers. Any product that may be evaluated in this article, or claim that may be made by its manufacturer, is not guaranteed or endorsed by the publisher.
